# Thermally induced crystallization, hole-transport, NLO and photovoltaic activity of a bis-diarylamine-based push-pull molecule

**DOI:** 10.1038/s41598-017-08606-6

**Published:** 2017-08-16

**Authors:** Yue Jiang, Magali Allain, Denis Gindre, Sylvie Dabos-Seignon, Philippe Blanchard, Clément Cabanetos, Jean Roncali

**Affiliations:** 1 Institute for Advanced Materials, South China Normal University, Guangzhou, 510006 China; 2 CNRS UMR 6200, MOLTECH-Anjou, University of Angers, 2 Bd Lavoisier, 49045 Angers, France

## Abstract

The synthesis of a molecule constituted of two diarylamine-based push-pull chromophores covalently linked *via* their nitrogen atom is described. Comparison of the electronic properties with the parent monomer shows that dimerization has negligible influence on the electronic properties of the molecule but exerts a dramatic impact on the capacity of the material to self-reorganize. Application of thermal annealing to thin films induces the crystallization under original morphologies, a process accompanied by a partial bleaching of the absorption in the visible range and by a huge increase of hole-mobility. X-ray diffraction data on single crystals reveal the presence of π-stacked organization with a non-centrosymmetric co-facial arrangement of the dipoles which leads to intrinsic 2^nd^ order bulk NLO properties of thin films as evidenced by second harmonic generation under 800 nm laser light. The implications of this thermally induced crystallization on the photovoltaic properties of the material are discussed on the basis of preliminary results obtained on simple bilayer organic solar cells.

## Introduction

**Figure 1:**
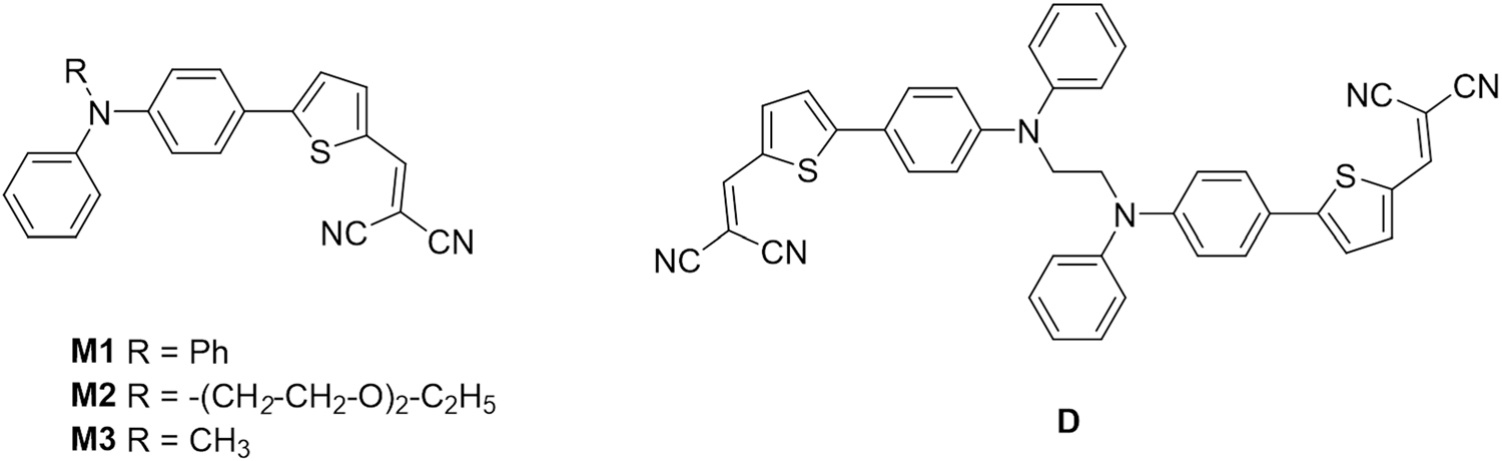
Chemical structure of push-pull monomers and of the target compound **D**.

Unquestionably, triphenylamine (TPA) represents a key building block for the preparation of active materials for organic light-emitting diodes (OLEDs)^1^, chromophores for dye-sensitized hybrid solar cells (DSSC)^2, 3^, organic photovoltaics (OPV)^4–7^ and more recently hole-transporting materials (HTM) for perovskite solar cells^8^. During the past decade, our group has synthesized various classes of donor materials for OPV taking advantage of the strong electron-donating properties and high hole-mobility of TPA-based materials and of a possible access to simple and cost-effective materials. For instance, promising conversion efficiencies have been reached with OPV cells based on the molecular donor **M1** ^9^ **(**Fig. 1) which can be prepared in only two steps at gram scale from low cost commercially available starting materials^10^.

Using **M1** as a reference compound, we have developed various structural modifications aiming at improving relevant photovoltaic parameters such as light-harvesting, open-circuit voltage or charge-transport^11–14^. In our continuing interest in materials based on simple structures and scalable syntheses^15^, we have undertaken an analysis of the effects of replacing one of the outer phenyl rings of the TPA block of **M1** by other aromatic groups^14^ or by aliphatic chains^16–18^. We have shown that although such substituents have a limited influence on the energy levels of the molecule, they can exert a considerable impact on the electronic properties of the resulting materials through the control of molecular organization. Thus, the introduction of a diether chain (**M2**) leads to a material that self-reorganizes in the solid-state into colorless crystalline films with mechanofluorochromic properties and intrinsic 2^nd^ order nonlinear optical properties^16^. In striking contrast, the introduction of a simple methyl group (**M3**) leads to a material which also undergoes solid-state self-reorganization but with a progressive decrease of the band gap, a fifty-fold increase of hole-mobility and a higher photovoltaic efficiency than **M1** ^17^. These intriguing phenomena clearly related to the substitution of the nitrogen atom of TPA, pose the question of the role of the substituent (nature, size, flexibility, hydrophilic/lipophilic balance etc.) on the self-organization of the material. Thus, in order to further investigate the structure-properties relationships in this class of materials, we report here on the synthesis and characterization of compound **D**, regarded as a dimer of **M3** in which the possibilities of self-reorganization are expected to be limited by the ethylene linker attached at the two nitrogen atoms.

## Results and Discussion

**Figure 2:**
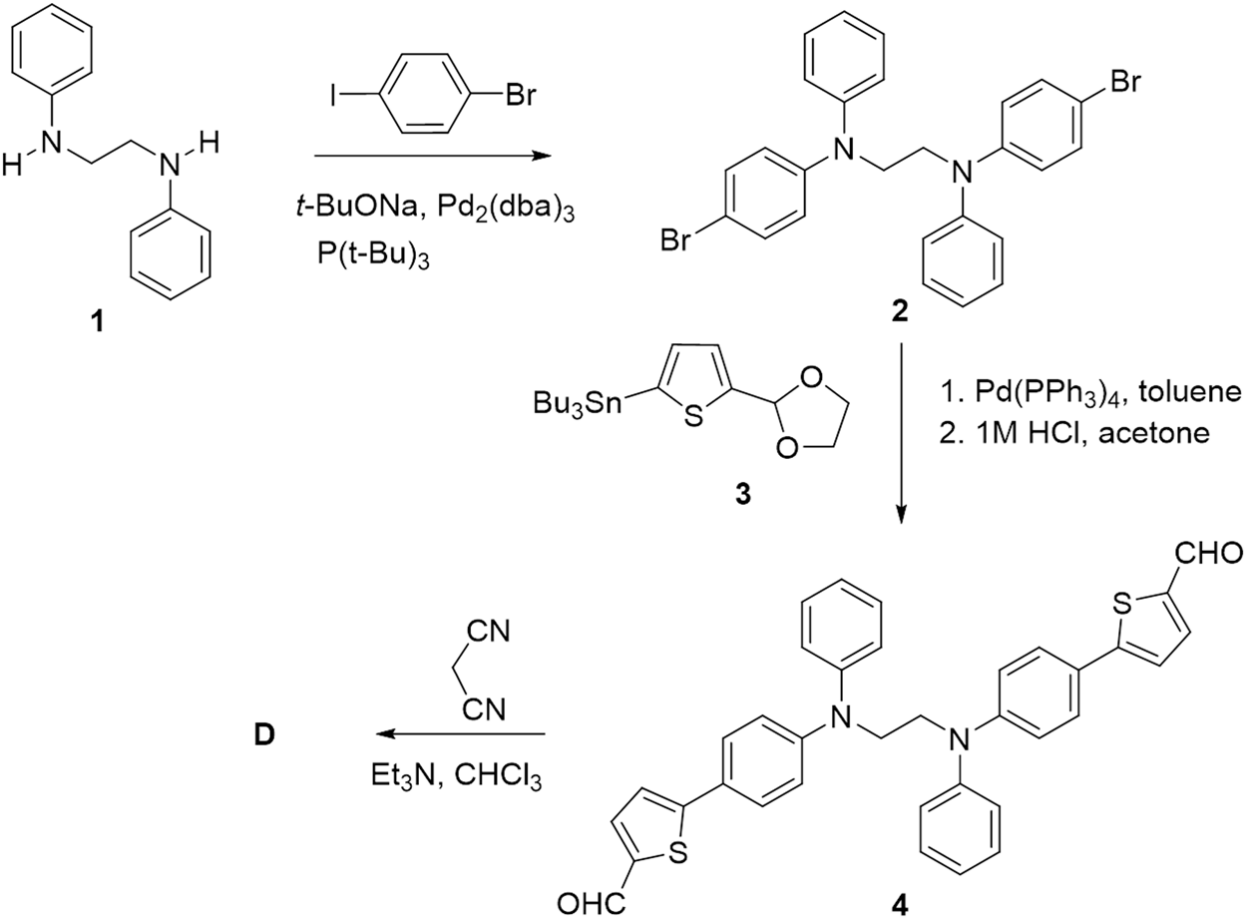
Synthesis of compound **D**.

The synthesis of D is depicted in Fig. 2. A double Buchwald-Hartwig cross-coupling reaction between the commercially available *N*,*N*-diphenylethane-1,2-diamine 1 and 1-bromo-4-iodobenzene gave the dibromo intermediate 2. This compound was subsequently engaged in a Stille cross-coupling reaction with (5-(1,3-dioxolan-2-yl)thiophen-2-yl)trimethylstannane 3^16^ leading, after deprotection, to dialdehyde 4. Finally, a Knoevenagel condensation of compound 4 with malonodinitrile in the presence of trimethylamine afforded the target molecule D as dark reddish powder.

The differential scanning calorimetry (DSC) trace of **D** shows a weak peak at 133 °C, followed by a huge endothermic melting peak at 261 °C suggesting a possible transition from the amorphous to a crystalline state followed by melting (Figure S1). Thermal gravimetric analysis (TGA) indicates a decomposition temperature of 350 °C. The much higher values of the melting and decomposition temperatures of **D** compared to the monomer **M3**, (respectively 261 *vs* 203 °C and 350 *vs* 277 °C) are consistent with the expected more restricted molecular freedom for the dimer.

**Figure 3:**
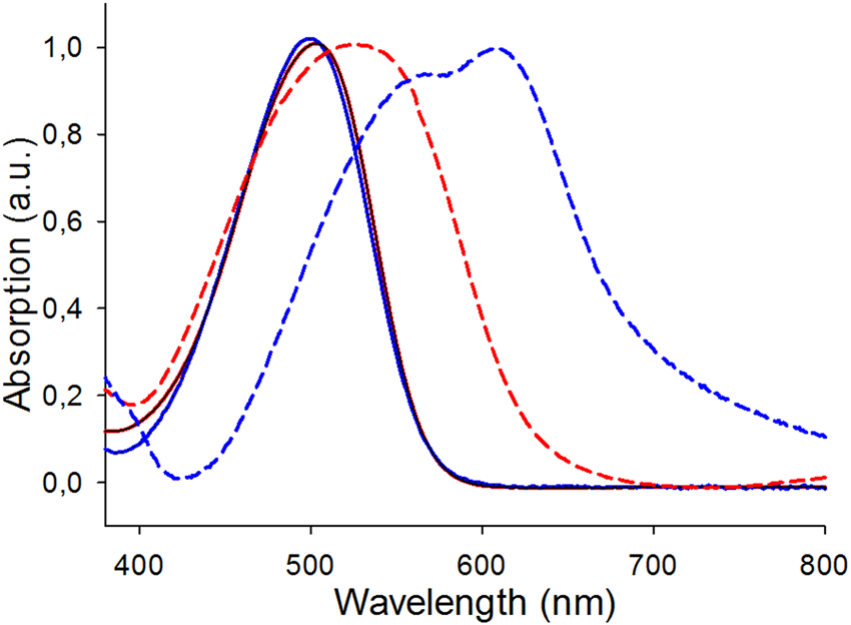
Normalized UV-vis absorption spectra of **M3** (blue) and **D** (red). In solution (solid lines) and as thin film on glass (dashed lines).

The UV-vis absorption spectrum of **D**, recorded in chloroform solution exhibits a broad absorption band with a maximum (*λ*_max_) at 502 nm (molecular extinction coefficient *ε* = 16500 M^−1^ cm^−1^) corresponding to an internal charge-transfer^4^. This spectrum is identical to that of the corresponding monomer **M3** (Fig. 3).

**Figure 4:**
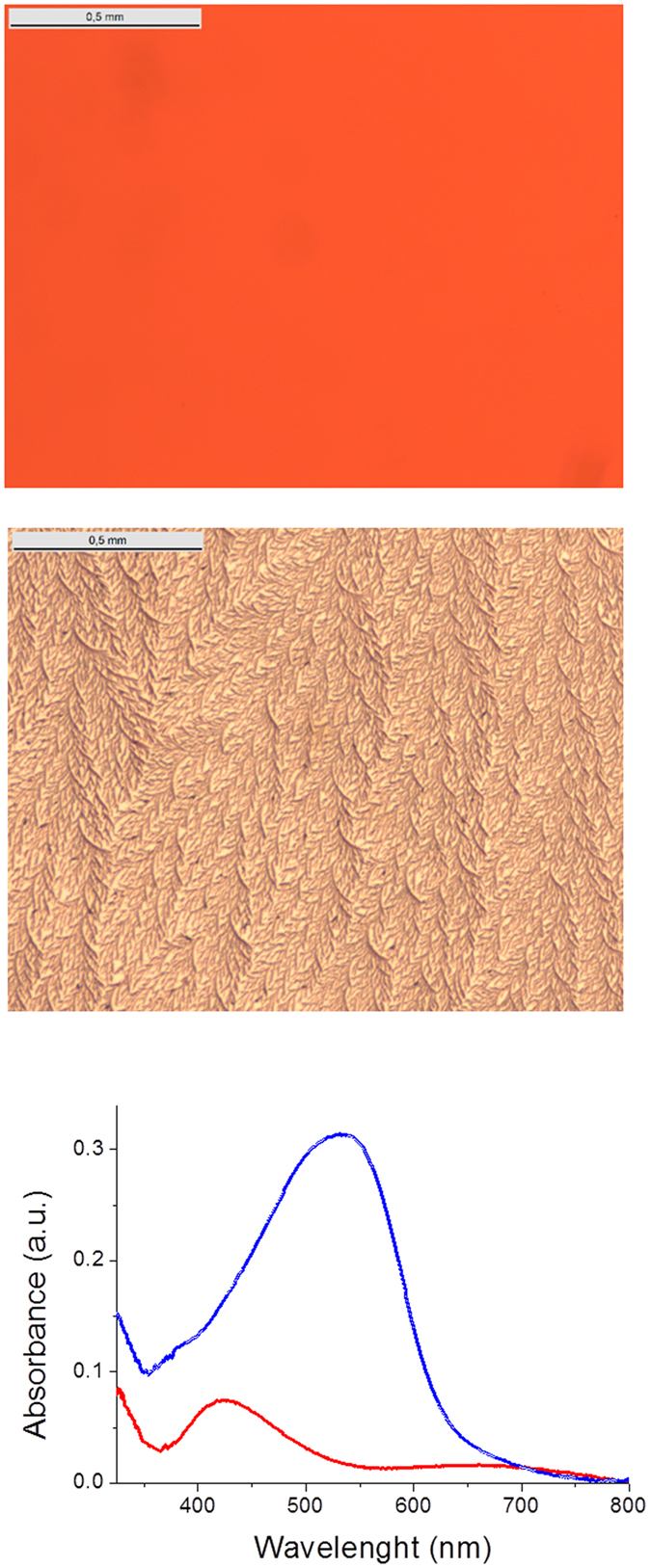
Top: Optical micrographs of as-deposited thin films of **D**; middle same film after 10 min at 140 °C; bottom UV-Vis absorption spectra of a film of **D** before (blue) and after (red) ten minutes at 140 °C.

When spun-cast on glass sheet, the spectrum of a thin film of **D** shows a broadened absorption band with a bathochromic shift of *λ*_max_ to 527 nm. The absorption onset at 630 nm leads to an estimated band gap (*E*_*g*_) of ~2 eV. Under the same conditions, the spectrum of films of **M3** shows a larger broadening of the absorption band, a red shift of *λ*_max_ to 607 nm and a band gap of 1.70 eV^17^. As already reported, while the very initial spectrum of films of **M3** is quite similar to that of films of **D**, within a few tens of minutes in ambient conditions, the material self-reorganizes leading to the low band gap spectrum shown in Fig. 4, a process which can be sped-up by a short thermal treatment (TT)^17^.

Figure 4 shows optical micrographs of a film of **D** before and after application of a 10 minutes TT at 140 °C namely slightly above the transition temperature observed in the DSC. While the initial film exhibits a smooth surface with a red colour, upon TT the colour changes to beige while the film presents a crumpled surface in which regular structures can be discerned. The corresponding UV-Vis absorption spectra show that TT produces a bleaching of the main absorption band in the 450-650 nm region leading to a final spectrum which presents a first band with a *λ*_max_ at 420 nm and a transition of low intensity around 630 nm (Fig. 4). These spectra suggest a change in molecular organization from random or *J*-type aggregates in the initial state to *H*-type aggregates in the annealed films. After application of TT the integrity of compound **D** was assessed and confirmed by mass-spectrometry, to rule out the hypothesis of decomposition. This process is very similar to that observed for films of the di-ether-substituted compound **M2** ^16^, however, a major difference is that for **M2** the process occurs spontaneously at room temperature whereas a TT at 140 °C is needed for **D**.

In order to gain more information on this thermally induced process, thin films of **D** prepared by vacuum deposition on PEDOT:PSS-coated ITO substrates have been analyzed by powder X-ray diffraction (XRD).

**Figure 5:**
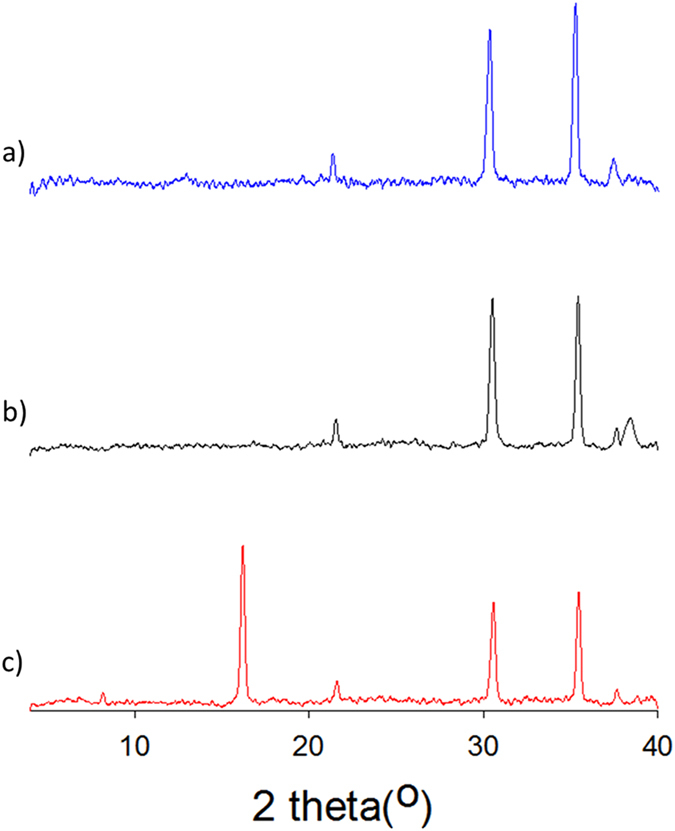
XRD patterns of (**a**) ITO/PEDOT:PSS substrate; (**b**)) same+ as deposited film of **D** (30 nm); (**c**) same after 5 min TT at 140 °C.

The XRD patterns in Fig. 5 show that in the absence of TT, no extra peak other than those observed for the reference substrates are detected, which is consistent with the amorphous nature of the film. However, heating the sample at 140 °C for 5 minutes produces the emergence of new peaks at 8° and 16° reflecting the tendency of the material to crystallize.

**Figure 6:**
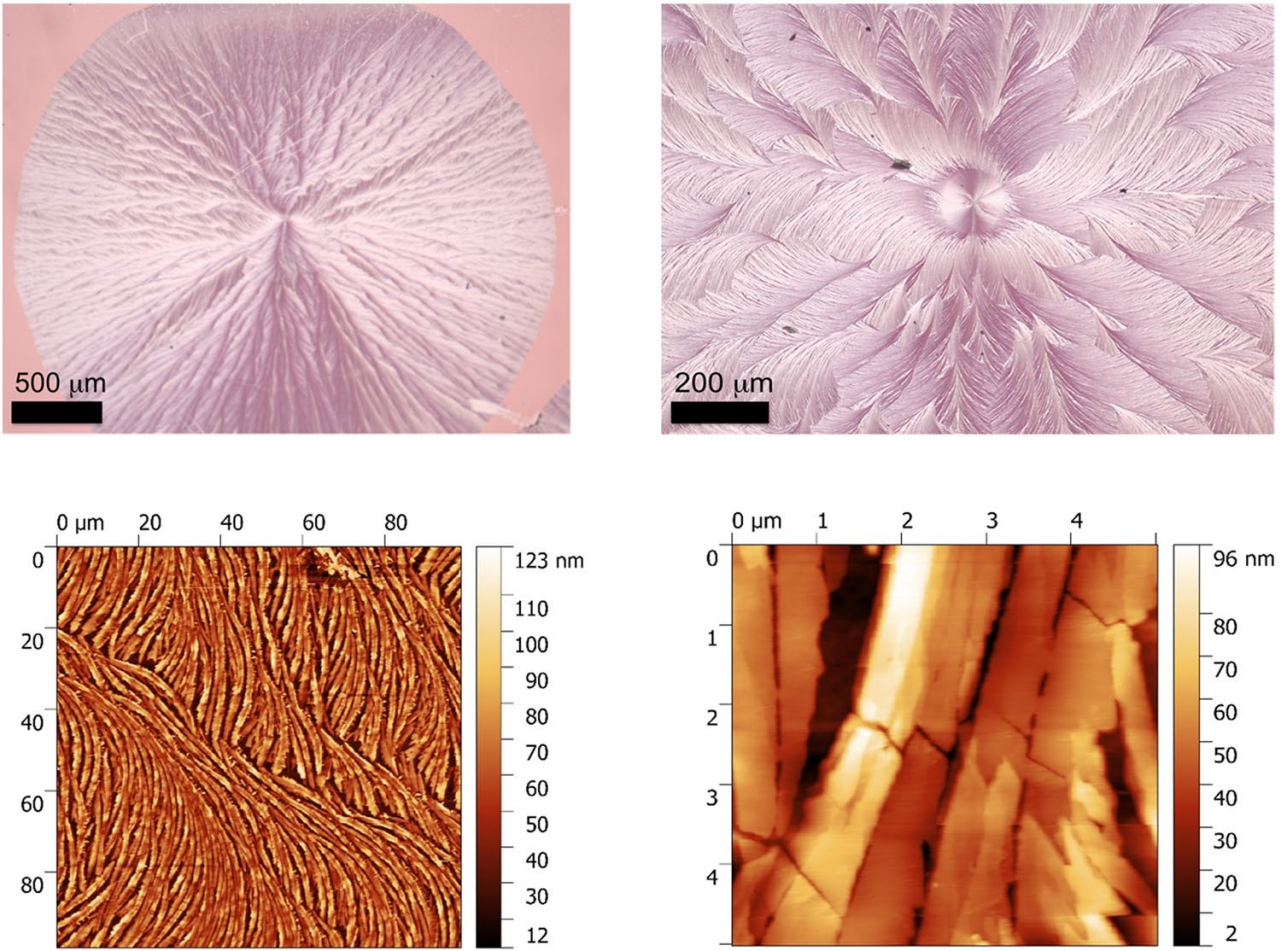
Top: optical micrographs of a thermally crystalized fiFClm of **D** on PEDOT:PSS coated ITO; bottom AFM images of the same film.

Further insights on the characteristics of this thermally induced crystallization (TIC) process are provided by examination of the surface morphology of the annealed films of **D** by optical microscopy. As appears in Figure 6, TT induces crystallization by spherical nucleation and growth. The images at higher magnification reveal well-defined feather-like patterns. Examination of these films by atomic force microscopy (AFM) shows that these patterns consist of micrometric crystalline fibrils of *ca* 500 nm diameter with lengths varying from a few microns to a few tenths of microns.

**Figure 7:**
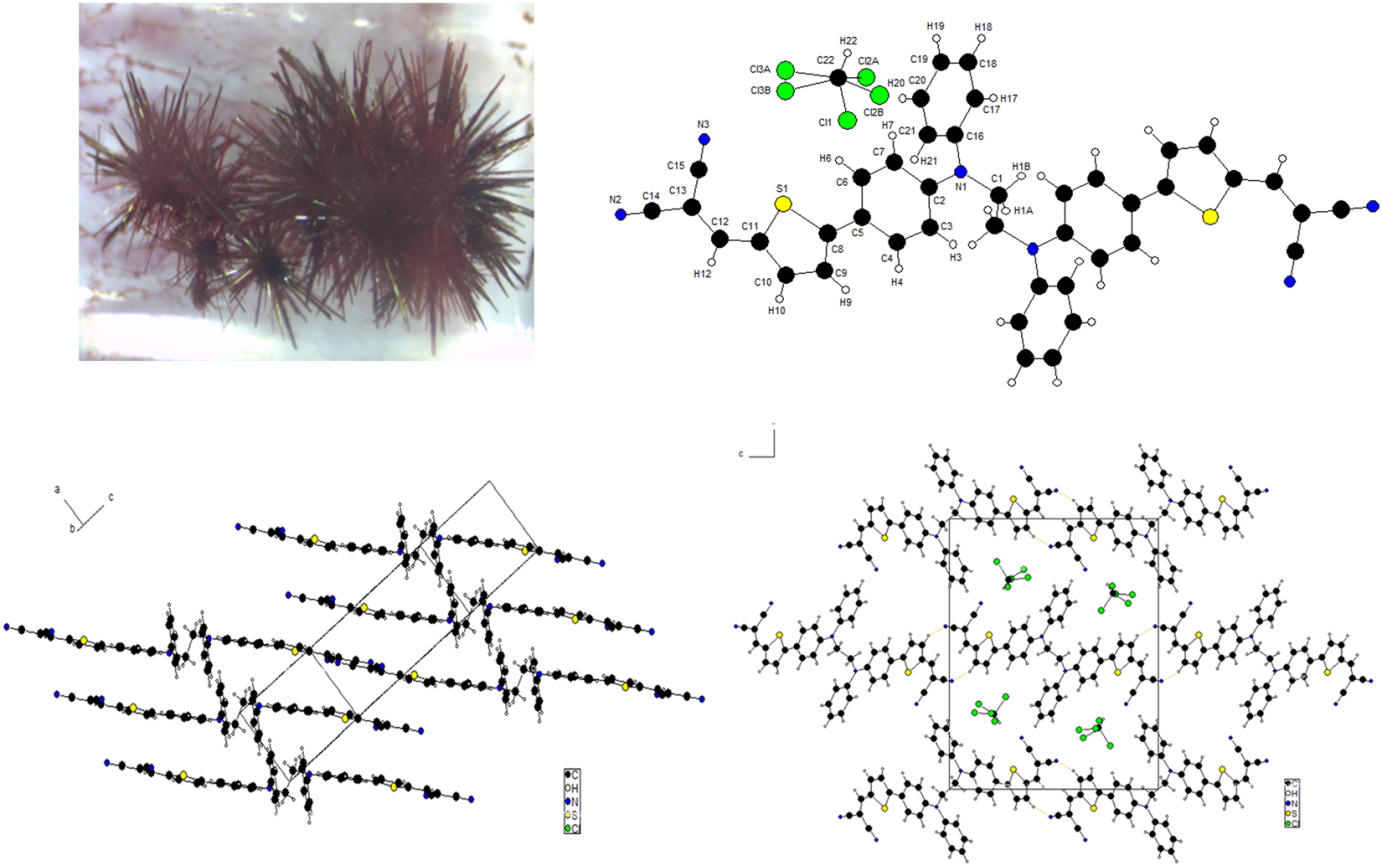
Top: Crystal (left) and molecular crystal structure of **D** (right). Bottom π-π stacking (left) and hydrogen bonding (right).

Slow evaporation of chloroform solutions of **D** leads to the formation of needle-like crystals which have been analyzed by X-ray diffraction^19^. As shown in Fig. 7 both sides of the molecule adopt the same quasi-planar conformation with angles of 5.449° between the inner benzene ring and the thiophene spacer and of 1.546° between the thiophene and the DCV group. On the other hand, the angle between the outer and the inner phenyl rings (75.6°) is larger than that observed for **M3** (69.0°)^17^. A well-organized π-π stacking is observed with an inter-layer distance of 3.4848 Å. Both parts of **D** align in a face-to-face organization as already observed for the monomer **M3**. Moreover, the outer phenyl rings also align in π-π stacking with distance of 3.0728 Å. Noticeably, hydrogen bonds (N2….H10 = 2.9903(23) Å) are observed between a nitrogen atom of the dicyanovinyl group and a β-hydrogen atom of the thiophene spacer of a neighbouring molecule forming tail-to-tail coplanar arrangements.

**Figure 8:**
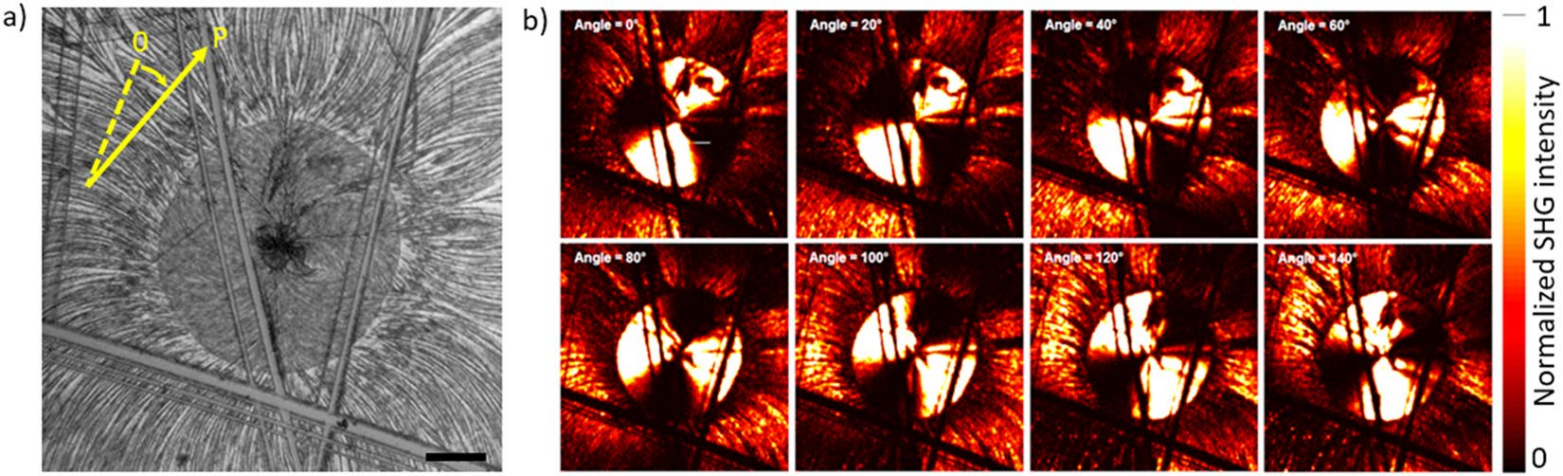
(**a**) Brightfield microscope view and (**b**) scans of transmitted SHG of the same part of a thermally crystallized film of **D** under irradiation with 800 nm laser light. The angle between the reference initial polarization (yellow dashed line in left) and the direction of linear polarization P of the laser for each SHG picture is indicated by yellow arrow. Incoming linear polarization is indicated in each picture and is rotating in steps of 20 deg. Scale bar: 100 μm.

We have recently reported that due to a highly dipolar structure and non-centrosymmetric arrangement of dipoles in the crystal thin films of **M2** exhibit spontaneous bulk 2^nd^ order NLO properties^16^. The presence of face-to-face dipoles in the crystal structure of **D** suggests that NLO properties could be expected in this case too. The 2^nd^ order NLO properties of the films of **D** were investigated by means of a polarization-dependent second harmonic generation (SHG) scanning microscopy setup (see SI). As appears in Fig. 8, irradiation of a part of a film, after TT, with 800 nm laser light produces intense SHG response at 400 nm. Molecular orientation anisotropy governs the NLO response as function of the direction of the linear polarization of the laser. On the other hand, the absence of SHG on non-annealed films under the same conditions confirms that the appearance of NLO properties is effectively related to the TIC of the material. Finally, it is noteworthy that this NLO activity still remains even after baking the sample at 140 °C in air, for more than a month, which confirms the high stability of the materials based on this class of molecules^18^.

In order to analyze the consequences of TIC on the charge-transport properties of **D**, the hole mobility has been measured by the space-charge-limited current method on hole-only devices before and after TT. To this end, thin films of **D** of *ca* 100 nm thickness have been thermally evaporated under vacuum on ITO/PEDOT:PSS substrates and gold electrodes were then deposited with the same method. In the absence of TT the films show a hole mobility (*μ*_H_) of 3.2 × 10^–8^ cm^2^ V^−1^ s^−1^, a value considerably inferior to that obtained with **M1** in the same conditions (1 × 10^–5^ cm^2^ V^−1^ s^−1^)^17^. However, application of a 30 min TT at 140 °C produces a huge increase of (*μ*_H_) which reaches a value of 1.0 × 10^–4^ cm^2^ V^−1^ s^−1^, thus confirming the strong impact of TIC on the charge-transport properties.

**Figure 9:**
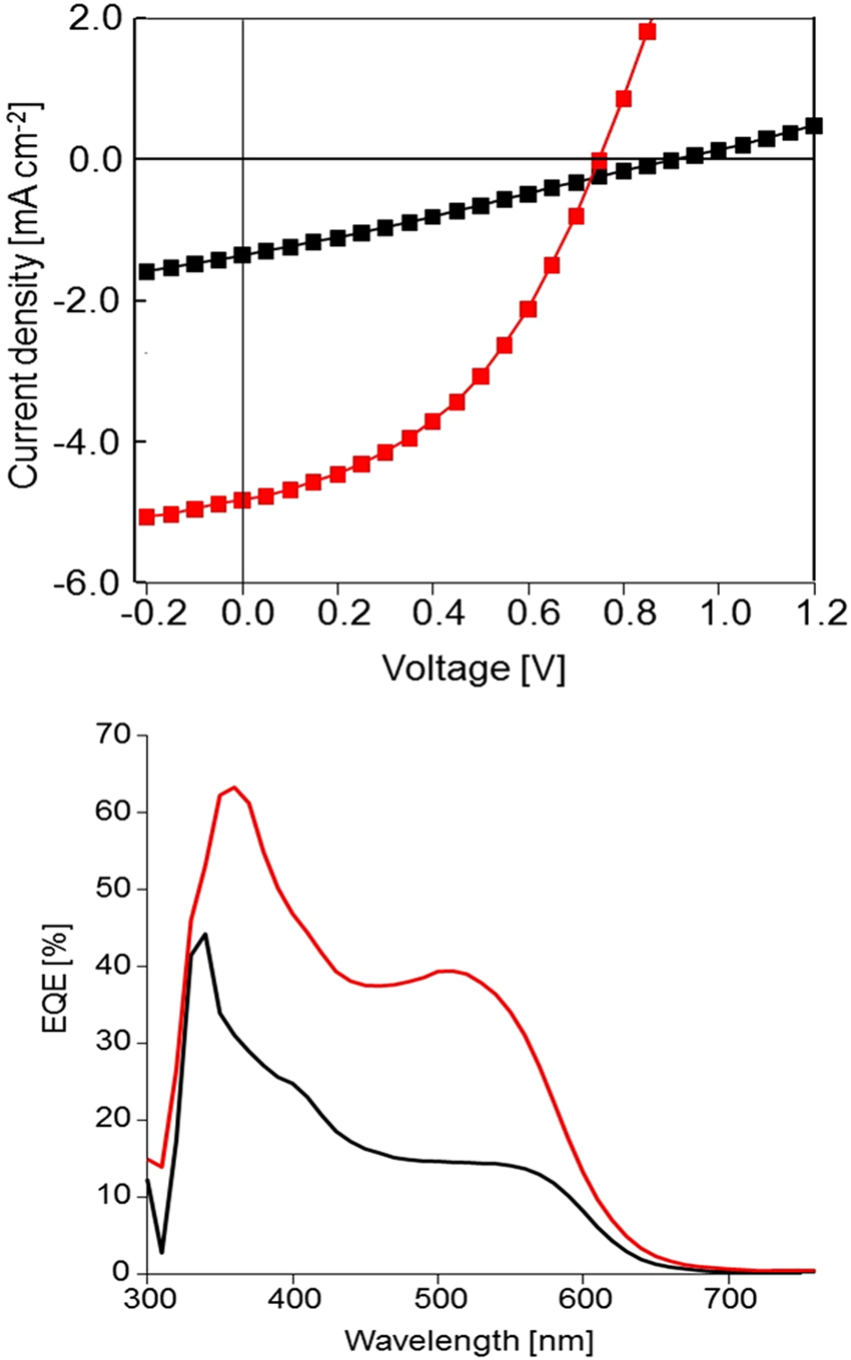
Top: Current density *vs* voltage curves of a bi-layer cell donor/C_60_ under AM 1.5 simulated solar light (80 mW cm^−2^). Black square: untreated cell, red squares: after 10 min TT at 140 °C. Bottom: *EQE* spectra of the same cell before (black) and after (red) TT.

As indicated in the introduction, the study of structure-properties relationships in this class of molecules was initially motivated by the development of simple active molecular materials for OPV. It was therefore interesting to investigate the effects of TT on the efficiency of **D** as donor material in OPV cells. The energy level of the HOMO and LUMO of **D** were estimated at *ca* −5.9 eV and −4.1 eV respectively from the onset of the oxidation and reduction waves recorded by cyclic voltammetry (see SI). The evaluation of the performances of **D** as donor material for OPV was carried out on simple bi-layer heterojunction solar cells fabricated by successive vacuum deposition of **D**, fullerene C_60_ and aluminium on ITO/PEDOT:PSS substrates. Figure 9 shows the current density *vs* voltage curves obtained under AM 1.5 simulated solar illumination. In the absence of TT, the cell delivers a short-circuit current density (*J*_*s*c_) of 1.36 mA cm^−2^ an open-circuit voltage (*V*_oc_) of 0.91 V and a fill factor (*FF*) of 0.27 leading to a *PCE* of 0.41% (Fig. 9 and Table 1). Application of a 5 min TT at 140 °C, produces an increase of *J*_*s*c_ to 4.82 mA cm^−2^ and a significant improvement of *FF*, leading to a five-fold increase of *PCE* to a value of ~2%.

**Table 1:** Hole mobilities measured on films and photovoltaic characteristics of bi-layer cells PEDOT:PSS/D/C_60_/Al under simulated AM 1.5 illumination at 80 mW cm^−2^ before (plain numbers) and after TIC (bold numbers).

*μ*_H_ [cm^2^ V^−1^ s^−1^]	*J*_sc_ [mA cm^−2^]	*V*_oc_ [V]	*FF*	*PCE* [%]
3.2 × 10^–8^	1.27	0.91	0.27	0.41
**1.0 × 10** ^**–4**^	**4.82**	**0.75**	**0.43**	**1.94**

The external quantum efficiency (*EQE*) spectrum of a non-treated cell recorded under monochromatic irradiation presents a first peak at 330–350 nm attributed to the combined contribution of C_60_ and of the arylamine block followed by a first shoulder around 400 nm and by a second broad shoulder extending to 650 nm corresponding to the ICT transition (Fig. 9). Application of TT produces an increase of the intensity of these various bands from 40 to 60% at 340–360 nm and 14 to 40% in the 510–550 nm region respectively. However, comparison of this *EQE* response to that obtained with **M3** ^17^ reveals a lower relative contribution of the ICT band for **D**. Taking into account the bleaching of the absorbance in the visible region produced by TT (Fig. 5), this suggests that the improvement of increase of *PCE* upon TT is due for a large part to the increase of hole mobility^20^, as already observed for other molecular donors^21^.

## Conclusion

A molecule constituted of two small arylamine-based chromophores covalently linked *via* their nitrogen atom has been synthesized. The analysis of the effects of TT of thin films by UV-Vis absorption spectroscopy, X-ray diffraction and optical and atomic force microscopies show that TT leads to a TIC associated with a transition from *J*- to *H*-aggregates, into ordered original structures. However, unlike some monomeric parent systems that undergo a similar process at ambient temperature, an additional input of thermal energy is required due to the restricted molecular freedom resulting from covalent dimerization. X-ray diffraction data of single crystals show the presence of co-facial arrangements of the dipoles which results in intrinsic 2^nd^ order bulk NLO properties for the crystallized films. Results obtained on “hole only” devices and bi-layer solar cells show that TIC strongly increases the hole-mobility and improves the photovoltaic conversion efficiency which however remains limited by a concomitant decrease of light-harvesting properties associated with TIC. To summarize these results confirm that the substitution of the nitrogen atom of arylamines represents an efficient approach for controlling the structure and electronic properties of the resulting materials. Research in this area is still at an early stage however, taking into account the key role or arylamine building blocks in the chemistry of advanced organic (opto)electronic materials, these results can be expected to stimulate further research in this direction.

## Electronic supplementary material

Supplementary info
