# Supplementary material for: Thermally induced crystallization, hole-transport, NLO and photovoltaic activity of a bis-diarylamine-based push-pull molecule
Source: Sci Rep. 2017 Aug 16;7:8317. doi: 10.1038/s41598-017-08606-6 (PMC5559478; doi:10.1038/s41598-017-08606-6)
Supplement: Supplementary file 1 — Supplementary info [file 41598_2017_8606_MOESM1_ESM.pdf]

Thermally induced crystallization, hole-transport, NLO and photovoltaic activity of a bis-diarylamine-based push-pull molecule

Yue Jiang, Magali Allain, Denis Gindre, Sylvie Dabos-Seignon, Philippe Blanchard, Clément Cabanetos, Jean Roncali

Supplementary information

General information

All reagents and chemicals from commercial sources were used without further purification. Solvents were dried and purified using standard techniques. Reactions were carried out under argon atmosphere unless otherwise stated. Chromatography was performed with analytical-grade solvents using Aldrich silica gel (technical grade, pore size 60 Å, 230-400 mesh particle size). NMR spectra were recorded with a Bruker AVANCE III 300 (^{1}H, 300 MHz and ^{13}C, 75 MHz). Chemical shifts are given in ppm relative to TMS and coupling constants *J* in Hz. IR spectra were recorded on a Bruker spectrometer Vertex 70 and UV-Vis spectra with a Perkin Elmer 950 spectrometer. Matrix Assisted Laser Desorption/Ionization was performed on MALDI-TOF MS BIFLEX III Bruker Daltonics spectrometer using dithranol as matrix.

Atomic force microscopy (AFM) experiments were performed using the Nano-Observer device from CSInstrument. The topographic images were obtained at room temperature in tapping mode. Images were processed with the Gwyddion free SPM data analysis software.

Synthesis

N-(4-bromophenyl)-N-(2-((4-bromophenyl)(phenyl)amino)ethyl)benzenamine (**2**): N,N′-diphenylethylenediamine **1** (2 g, 9.43 mmol), 1-bromo-4-iodobenzene (5.33 g, 18.86 mmol), sodium tert-butoxide (2.72 g, 28.29 mmol) and tris(dibenzylideneacetone)dipalladium(0) (338 mg, 0.37 mmol) were poured in a 250 mL flask and dried overnight under vacuum. Powders were then solubilized in dry toluene (110 mL), and the resulting mixture was degassed for 30 min by argon bubbling before adding tert-butylphosphine (1M in toluene, 2.26 mL, 2.26 mmol). After a night of stirring at 50°C, the reaction mixture was quenched by distilled water and extracted with diethyl ether. The crude was finally purified by silica gel chromatography using petroleum: dichloromethane in a 4:1 ratio to afford **2** as a white powder (530 mg, 11%). ^{1}H NMR (300 MHz, $CDCl_3$) δ = 3.98 (2H, s), 6.75 (2H, d, *J*=9.05 Hz), 7.00 (2H, d, *J*=8.8 Hz), 7.05 (1H, t, *J*=X.X Hz), 7.30 (2H, m). ^{13}C NMR (76 MHz, $CDCl_3$) δ = 49.74, 112.97, 120.94, 122.19, 122.83, 129.62, 132.15, 146.83.

5,5'-(4,4'-(ethane-1,2-diylbis(phenylazanediyl))bis(4,1-phenylene))dithiophene-2-carbaldehyde (4): Compounds **2** (530 mg, 1.02 mmol) and **3** (979 mg, 3.06 mmol) were solubilized in dry toluene (100 mL), preliminary degassed for 30 min. Then, tetrakis(triphenylphosphine)palladium(0) (120 mg, 0.1 mmol) was added and the reaction mixture was refluxed overnight at 120°C. After the consumption of the starting material, reaction was cooled down to room temperature and quenched by distilled water. Extracted with diethyl ether, the crude was purified by silica gel chromatography using a mixture of petroleum ether and dichloromethane (1:5). Once concentrated, the compound was dissolved in acetone (100 mL) and treated with 1M HCl (100 mL) and stirred overnight at room temperature in order to generate the aldehyde groups. Finally, an orange fluffy solid (150 mg, 25%)

was obtained by extraction with diethyl ether, evaporation and purification on silica gel using pure dichloromethane as the eluent. ^{1}H NMR (300 MHz, $CDCl_3$) δ = 4.10 (2H, s), 6.81 (2H, d, *J*=9.1 Hz), 7.14 (2H, d, *J*=7.9 Hz), 7.20 (1H, t), 7.30 (1H, d, J=4.0 Hz), 7.39 (2H, t), 7.52 (2H, d, *J*=9.0 Hz), 7.73 (1H, d, J=4.2 Hz), 9.88 (1H, s). ^{13}C NMR (76 MHz, $CDCl_3$) δ = 49.71, 116.79, 122.35, 123.99, 124.72, 124.87, 127.51, 129.93, 137.91, 140.90, 146.27, 149.00, 154.96, 182.59. HRMS-MALDI-TOF calculated for 584.2, found 584.2.

2,2'-(5,5'-(4,4'-(ethane-1,2-diylbis(phenylazanediyl))bis(4,1-phenylene))bis(thiophene-5,2-diyl))bis(methan-1-yl-1-ylidene)dimalononitrile (D): Compound **4** (210 mg, 0.19 mmol) and malononitrile (50 mg, 0.76 mmol) were dissolved in 50 mL of chloroform before adding two drops of trimethylamine. After a night of stirring at room temperature, the precipitated red solid product was filtered and washed with dichloromethane (100 mg, 85%). ^{1}H NMR (300 MHz, $CDCl_3$) δ = 4.09 (2H, s), 6.81 (2H, d, *J*=8.5 Hz), 7.13 (2H, d, *J*=8.6 Hz), 7.21 (1H, t), 7.36-7.40 (2H, t), 7.62-7.67 (3H, t), 7.90 (1H, d, J=4.2 Hz), 8.58 (1H, s). ^{13}C NMR (126 MHz, DMSO, 70°C) δ = 156.58, 152.27, 149.79, 146.08, 142.56, 132.74, 130.02, 128.00, 125.17, 125.05, 123.91, 122.73, 116.79, 115.05, 114.34, 73.07, 49.92. HRMS-MALDI-TOF calculated for 680.1817, found 680.1809. Melting point: 261°C.

Fabrication and characterization of solar cells

Indium-tin oxide coated glass slides of 24 × 25 × 1.1 mm with a sheet resistance of RS = 7 Ω/□ were purchased from Praezisions Glas & Optik GmbH. The ITO layer was patterned *via* a 37% hydrochloric acid solution and zinc powder etching. The substrates were then washed with a diluted Deconex® 12 PA-x solution (2% in water) under ultrasound for 15 min and scrubbed using dishwashing soap before being cleaned by a series of ultrasonic treatments for 15 min in distilled water (15.3 MΩ cm^{-1}), acetone and isopropanol. Once dried under a steam of nitrogen, a UV-ozone plasma treatment (UV/Ozone ProCleaner Plus, Bioforce Nanosciences) was performed for 15 min. A filtered aqueous solution of poly(3,4-ethylenedioxy-thiophene)-poly(styrenesulfonate) (PEDOT:PSS; Clevios P VP. AI 4083) through a 0.45 μm PTFE membrane (Millex®) was spun-cast onto the patterned ITO surface at 5000 rpm for 40 s before beeing baked at 140 °C for 15 min leading to a *ca* 40 nm layer of PEDOT-PSS.Films of **D** (15 nm) were then prepared by thermal evaporation under vacuum (*ca* 10^{-6}mbar using a Plassys Bestek ME300 evaporator). Finally, 20 nm of C_{60} fullerene (99+%, MER Corporation) and 150 nm of aluminum were successively and thermally evaporated on top of the donor layer through a shadow mask defining six cells of 27 mm^2 each (13.5 mm x 2 mm). *J vs V* curves were recorded under illumination using a Keithley 236 source-measure unit and a home-made acquisition program. The light source is an AM1.5 Solar Constant 575 PV simulator (Steuernagel Lichttecknik, equipped with a metal halogen lamp). The light intensity was measured by a broad-band power meter (13PEM001, Melles Griot).The External Quantum Efficiency (EQE) spectra were recorded under ambient atmosphere using a halogen lamp (Osram) with an Action Spectra Pro 150 monochromator, a lock-in amplifier (Perkin-Elmer 7225) and a S2281 photodiode (Hamamatsu).

Determination of hole-mobility

Thin films of various thicknesses were prepared by vacuum deposition of **D** on the above described PEDOT:PSS substrates. Gold electrodes (150 nm) were then thermally evaporated under a vacuum of 1.5 x 10^{-5} Torr, through a shadow mask defining actives area of 12.60 mm^2, 3.10 mm^2 and 0.78 mm^2 per substrates. Hole mobilities (μ_h) were evaluated by the spece-charge

limited current method using the Mott-Gurney law, *ie*, $J_{SCLC} = (9/8)\varepsilon_r\mu_h(V^2/D^3)$ where ε_r is the static dielectric constantof the medium ($\varepsilon_r = 3$) and d, the thickness of the active layer.

Thermal analyses

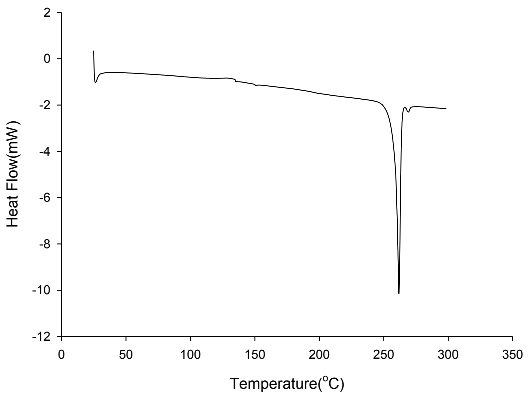

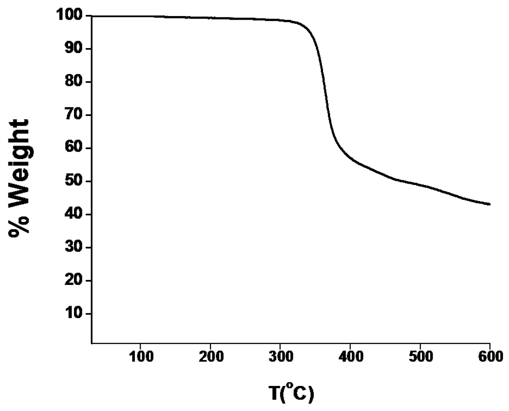

Figure S1. Traces of DSC (top) and TGA (bottom) for **D**, recorded under nitrogen at a scan rate of 10°C/ min.

Cyclic voltammetry was performed in 0.10 M Bu_4NPF_6/CH_2Cl_2 (HPLC grade). Solutions were degassed by nitrogen bubbling prior to each experiment. Experiments were carried out in a one-compartment cell equipped with platinum electrodes and a saturated calomel reference electrode (SCE) using a Biologic SP-150 potentiostat with positive feedback compensation

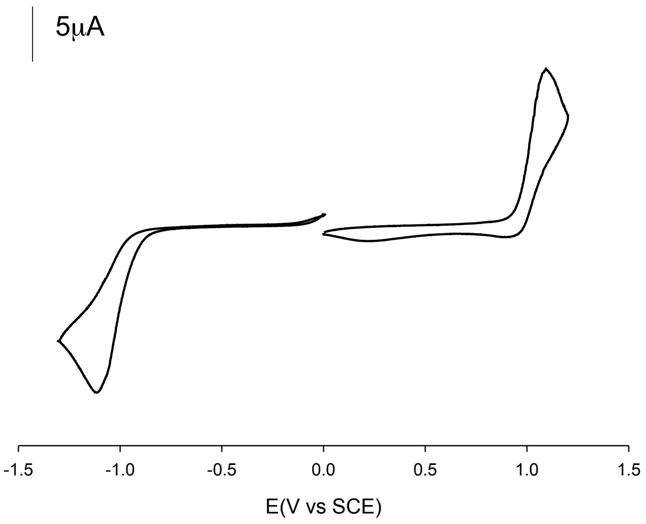

Fig. S2 Cyclic Voltammetry of D, 1mM in 0.10M Bu_4NPF_6/CH_2Cl_2, scan rate 100mV s^{-1}, Pt working electrode.

The energy level of the HOMO and LUMO of D were estimated at *ca* – 5.9 eV and – 4.1 eV respectively from the onset of the oxidation and reduction waves recorded by cyclic voltammetry

Crystallographic structure

X-ray single-crystal diffraction data for **D** were collected at 200K on an Agilent Technologies SuperNova diffractometer equipped with Atlas CCD detector and mirror monochromated micro-focus Cu-K_α radiation (λ = 1.54184 Å). The structure was solved by direct methods and refined on F^2 by full matrix least-squares techniques using SHELX97 programs (G.M. Sheldrick, 1998). All non-H atoms were refined anisotropically and the H atoms were included in the calculation without refinement. Multiscan empirical absorption was corrected using CrysAlisPro program (CrysAlisPro, Agilent Technologies, V1.171.37.35g, 2014). Inside the structure, the chloroform solvent is statistically disordered and 2 Cl are refined on two positions.

Crystallographic data for **D** : $C_{44}H_{30}Cl_6N_6S_2$, M = 919.56, red needle, 0.328 x 0.078 x 0.049 mm^3, monoclinic, space group $P2_1/n$, a = 5.5436(1) Å, b = 21.9013(3) Å, c = 17.7862(2) Å, β = 97.650(1)°, V = 2140.24(5) $Å^3$, Z = 2, ρcalc = 1.427 g/cm^3, μ(CuKα) = 4.896 mm^{-1}, F(000) = 940, θmin = 3.22°, θmax = 76.45°, 10591 reflections collected, 4361 unique (R_{int} = 0.021), parameters / restraints = 270 / 0, R1 = 0.0445 and wR2 = 0.1224 using 3801 reflections with $I>2\sigma(I)$, R1 = 0.0518 and wR2 = 0.1300 using all data, GOF = 1.045, $-0.603 < \Delta\rho < 0.318$ e.$Å^{-3}$. CCDC-1531046 contains the supplementary crystallographic data for this paper.

Polarization-dependent Second Harmonic Generation scanning microscopy experiments.

The 2_{nd} order NLO properties of the materials was investigated by means the SHG scanning microscopy setup (Figure S2) which can provide 2D (thin films) or 3D (bulk materials) mapping of the SHG signal. The laser beam (pulse duration 100 fs, repetition rate 80 MHz) is focused and moved on the sample by means of two galvanometric mirrors, while the SHG signal is detected with of a photon counter coupled with a fast photomultiplier. The laser beam scans a selected area on the

sample, while for every position of the focused beam, the SHG signal is acquired and stored to finally produce 1 pixel of the image and finally provide the SHG image (200x200 pixels). The laser power has been adjusted to 120 mW, the laser wavelength was 800 nm and the pixeltime was 20 µsec.

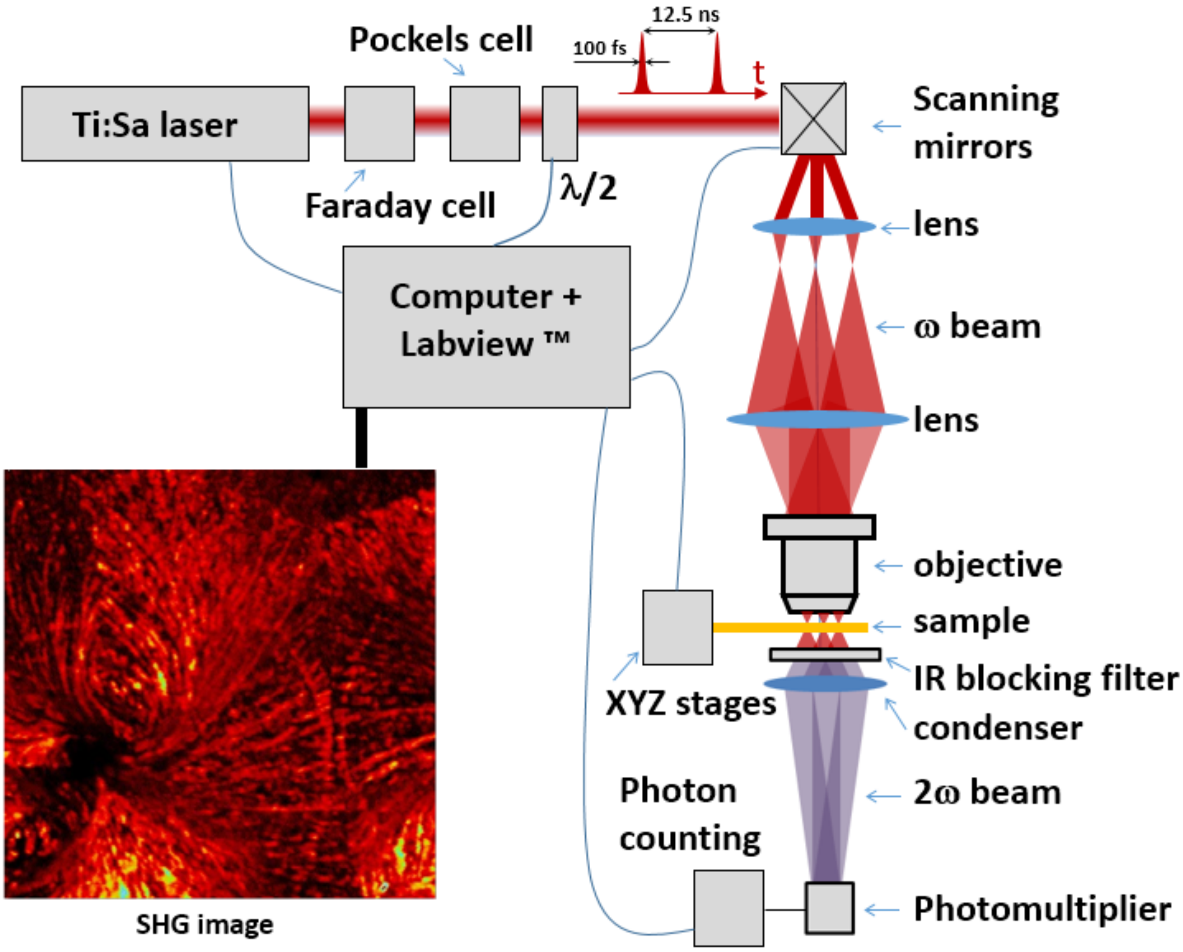

Figure S3. Experimental set up for SHG scanning microscopy experiments.

The laser source is a Ti:Sapphire (Tsunami, Spectra Physics). The pump laser is a 10 W solid-state laser (Millenia Xs, Spectra Physics). The angular deviation of the beam was controlled with an X-Y scanner (Cambridge Technology). The laser power was adjusted by means of an electro-optic modulator (Pockels cell 350-80LA, Conoptics). A Faraday rotator (IO-3-780-HP, Thorlabs) was used as an optical isolator. An achromatic half-wave plate (700-1000 nm, Edmund Optics) placed on a motorized rotating stage (PR50, Newport) is used to control the polarization of the incident beam. The beam was focused on the sample with an x20 objective lens (Olympus, N.A. 0.25). The SHG signal selection is realised with a Notch filter centered on 808 nm intended to absorb the excitation beam (NF808-34, Thorlabs) and followed by a bandpass filter with a bandwith of 40 nm and central transmitted wavelength of 400 nm (FBH400-40, Thorlabs). The SHG photons are detected by a photomultiplier (H744P-40, Hamamatsu) coupled with a photon counter (C9744, Hamamatsu). The glass slide where the sample was deposited is placed on XYZ motorized stages (Newport) controlled by an 8 axes motion controller/ Driver (XPS, Newport). The light polarization, the voltage control of the galvanometric mirrors and the micropositioning of the sample are precisely piloted by means of a

homemade National Instrument Labview™ program. This program is used to store the signal for each position of the scanned laser beam on the sample, to provide finally the SHG images.
